# A Measurement-Aided Control System for Stabilization of the Real-Life Stewart Platform

**DOI:** 10.3390/s22197271

**Published:** 2022-09-26

**Authors:** Wojciech P. Hunek, Paweł Majewski, Jarosław Zygarlicki, Łukasz Nagi, Dariusz Zmarzły, Roman Wiench, Paweł Młotek, Piotr Warmuzek

**Affiliations:** 1 Faculty of Electrical Engineering, Automatic Control and Informatics, Opole University of Technology, Prószkowska 76 Street, 45-758 Opole, Poland; 2 MovieBird International, Romualda Traugutta 5 Street, 45-667 Opole, Poland

**Keywords:** Stewart platform, measurement system, stabilization, control system, practical implementation

## Abstract

In the paper, an innovative control system devoted to the stabilization of the parallel manipulator-type Hexapod is presented. The device consists of three main parts, allowing us to reach the desired location during various external disturbances. Indeed, the telescopic boom located on the car along with the system providing the correction of the boom column deflection as well as the gyroscopic self-leveling head constitute a complex tool covering a plethora of modern techniques and solutions. Through the application of advanced issues strictly derived from nonlinear identification and multivariable control theory branches, the dynamical behavior of the discussed device has been handled in order to achieve a proper reference operation. Naturally, it has been supported by a set of accompanying approaches related to the processes of the real-time measurement and robust data transmission. It should be emphasized that the proposed computer-aided system is intended for the film industry, where image stabilization plays a crucial role. Such a statement has additionally been confirmed by other innovative products introduced by a company placed in Opole, Poland, called MovieBird International.

## 1. Introduction

In the literature, we can find a number of articles concerning the stabilization of position and tilt observed during crane boom movement [1,2,3,4,5]. The reason for this is a great interest in the mentioned technology due to the high implementability in various fields, mainly covering the film industry [6,7,8]. On the other hand, the discussed technology corresponds to a telescopic crane that can be treated as a complex mechanical system from the control theory and practice point of view. Its dynamic model is strongly nonlinear and should be classified as the underactuated one [9,10,11,12]. It should be emphasized that a plethora of studies are concerned with the application of control methods that enable the minimization of swinging and swaying as well as tilting of cranes. For example, Wu et al. [11] have developed a nonlinear model considering the output signals from five encoders. In order to have efficient control, they used an adaptive algorithm based on the fuzzy logic paradigm [13,14,15]. The model allows us to minimize some external disturbances deriving from wind gusts and nonlinear drawbacks in relation with dead-zones of mechanical components. The stability of the equilibrium point of the discussed nonlinear boom model has been examined using the commonly known Lyapunov method. On the other hand, Fujita and Sugiyama [16] have proposed a telescopic boom model that has taken into account the shifting nodal coordinates under the assumption of shifting common constraints. Additionally, it has covered the flexibility of the beams that have formed the boom. Nonlinear effects caused by both the dynamics of sliding contacts and the friction process have also been considered here. Moreover, Koumboulis et al. [17] have presented a mathematical model of a nonlinear tower crane in the state-space framework. They have used the ZigBee protocol-originated wireless sensors to gather the current position of the crane. In order to position control, a network of regulators based on the PID controllers has additionally been investigated.

However, it should be emphasized that most studies are concerned with stationary booms. A small amount of research has been conducted by employing mobile booms. For instance, Pertsch and Sawodny [18] have explored vibration mitigation of telescopic ladders in fire department vehicles. The authors have proposed a model respecting the coupling of bending and torsional forces. The model has allowed us to include both vertical boom slope and section asymmetry. In order to measure tilt of the boom, the gyroscope located at the top of the device has been used. An active vibration suppression algorithm has additionally been suggested, employing the Luenberger observer.

Taking into account the aforementioned considerations, it should strongly be emphasized that there are no studies in the literature covering the stabilization of telescopic booms in motion. However, the discussed issue has partially been addressed in the work of Kalmari et al. [19]. The authors have investigated the feasibility of an active tilt reduction in the articulated boom observed in a wood cutting and handling machine. A set of two gyroscopes and accelerometers have been applied to study the position and inclination of the operating tools. The rotation angle and angular velocities of the tools have been estimated using an extended Kalman filter. A similar idea is also examined while working the cranes on the open sea. For example, Ismail et al. [20] have described the dynamics of a ship-mounted crane system, which has respected the disturbances induced by ocean waves and strong winds. For the purpose of regulation, a controller based on the linear-quadratic algorithm has been proposed.

Concluding, the touched problem has a scientific and application-oriented nature. This brief overview shows that the proposed mobile technology falls within the area of pending issues. This is due to the fact that the addressed boom model is highly nonlinear, and its description requires the application of complex mathematical approaches. A group of important issues to be solved also complements the crucial control, which expects advanced algorithms operating in real time. The implementation of the discussed solution can be treated as the innovative one, which has never been seen before. There are no similar methods to that provided by the article, what can be treated as a significant extension of the existing ideas, especially in terms of their reasonable combination. The main contributions of the paper are as follows:

- An original measurement-aided approach to the stabilization of the physical Stewart platform system is introduced.
- A proposed algorithm is strictly dedicated to the complex nonlinear plant subjected to a set of different disturbances.
- A smart methodology employing the innovative embedded-originated sensor and control layers is accented.
- A number of analytical investigations involving the signal processing-based operations mainly respecting the Lagrangian and Kalman paradigms are considered.
- The real-life confirmation certainly favors an application of the Stewart platform stabilization system in the modern film industry.

The paper is organized in the following manner. Section 2 describes in detail the examined device. Subsequently, in Section 3, an idea of the measurement system is shown, in particular, from the practical point of view. The crucial control is proposed in Section 4. The summary along with open problems successfully summarizes the achievements of the paper.

## 2. System Representation

**Figure 1:**
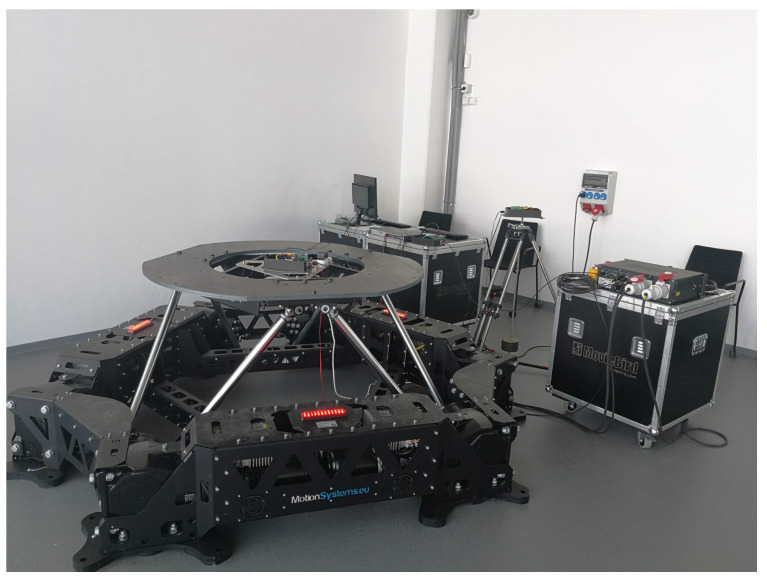
Real-life Stewart platform system: PS-6TM-2500 [Source: MovieBird Intl.].

**Figure 2:**
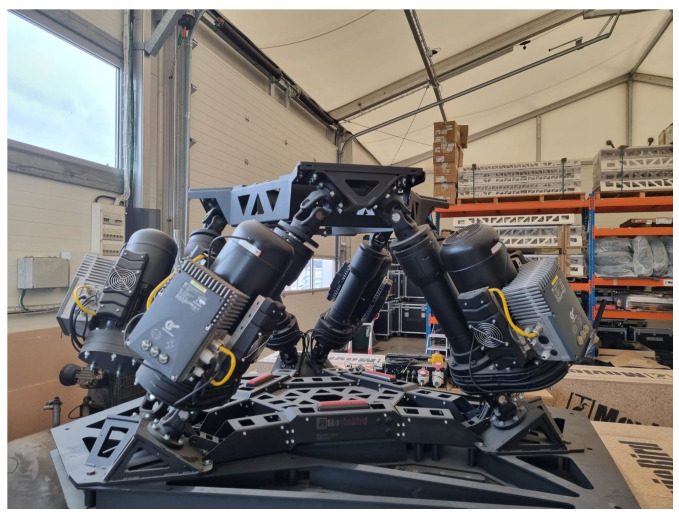
A real-life compensation system involving two Stewart platforms [Source: MovieBird Intl.].

In order to measure and stabilize in a real-time process, the physical Stewart platform system is examined, as shown in Figure 1 [21]. Additionally, the second real-life plant is applied to compensate the dynamic behavior only in the laboratory case (see Figure 2). Indeed, the proposed solution allows us to predefine a wide range of results before putting the entire complex object on the devoted vehicle.

Observe that following a notion of Refs. [22,23], the commonly known six degrees of freedom-originated Stewart platform system derived from the Lagrangian paradigm can be described in the comprehensive manner. Thus, from the Lagrangian method, we obtain for the chosen linearized operating point

(1) L=T−U,

where

(2) $$T=\frac{1}{2}mv^{2}+\frac{1}{2}I\omega^{2},$$

and

(3) U=mgh,

stand for the kinetic and potential energies, respectively, whereas the symbols *m*, *v*, *I*, ω, *g* and *h* denote the corresponding mass, velocity, inertia moment, angular velocity, gravity and altitude.

Now, respecting the Euler–Lagrange equation, expressed as follows [24,25,26,27,28,29,30]

(4) $$\frac{d}{dt}\left(\frac{\partial L}{\partial \dot{q}}\right)-\frac{\partial L}{\partial q}+\frac{\partial F}{\partial \dot{q}}=Q,$$

where parameters *q*, *F* and *Q* are the vector of generalized coordinates, friction and damper forces vector as well as the generalized forces vector, respectively, combined with the discussed motion formula, the overall dynamic equation can be formed in the following manner

(5) $$M\left(q\right)\ddot{q}+C\left(q,\dot{q}\right)\dot{q}+G\left(q\right)+F\left(\dot{q}\right)=\tau .$$

Observe that the crucial relation (5) employs the parameters $M\in R^{6\times 6}$, $C\in R^{6\times 6}$, $G\in R^{6\times 1}$, *F* and τ being the inertia matrix, Coriolis/centripetal matrix, vector of the gravity expressions, friction element and a reported torque vector. Naturally, the explained approach has a number of useful properties, for instance see Refs. [31,32,33].

Having the fundamental notions covering the control object, let us switch to present the crucial measurement system applied in order to gather the process data.

## 3. Measurement System

**Figure 3:**
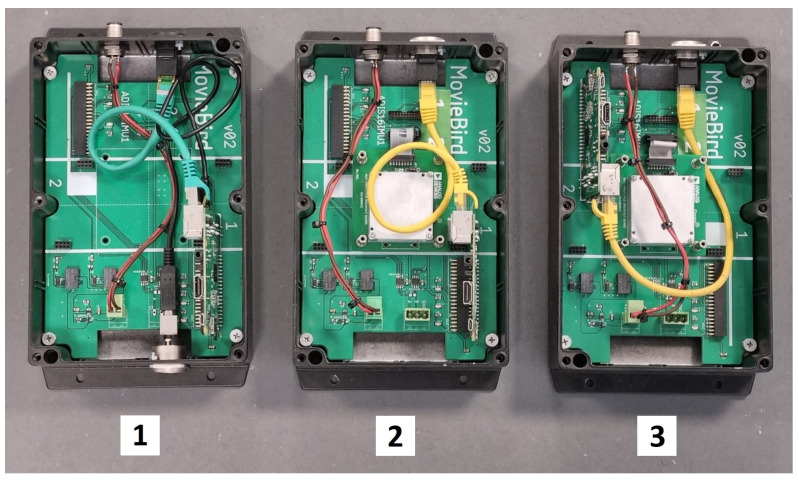
Core modules of the developed stabilization system: 1—server element, 2—accelerometer element, 3—main gyroscope sensor element [Source: MovieBird Intl.].

For the purpose of employing the stabilization system, the entire structure of the measurement and control tasks was successfully designed. For that reason, the three Raspberry Pi-oriented original model computers were proposed and implemented using the original PCB modules (see Figure 3). The discussed solution allows us to arrange the structures of circuits and place the sensitive electronics in the waterproof boxes.

**Figure 4:**
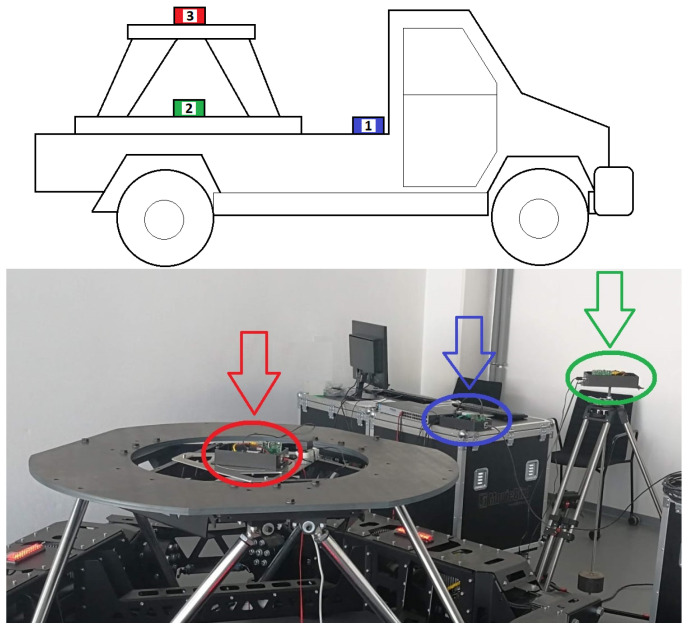
A module configuration (colored shapes): vehicle vs. laboratory instances [Source: MovieBird Intl.].

Going further, the components of the complex system of Figure 3 were directly connected through the one network engaging the commonly known UDP protocol. Naturally, the chosen number of modules results from the nature of the investigated process, covering additional parameters derived from the dynamics of the plant. In fact, the Stewart stabilization platform is intended to take place on a moving vehicle; therefore, apart from topology, the structural arrangement has great importance as well (see Figure 4).

Following the correct overall process, the measurement system was directly located on a moving car. Thus, the problem of stabilization is not trivial, and the additional disturbances in the form of vehicle accelerations should be respected. For that reason, the original configuration was assumed. The crucial sensor module, certainly applied during the stabilization action, was placed on the base of the upper Stewart platform (red color—No. 3). Moreover, the attached-to-the-car additional accelerometer element allows us to measure the disturbances in the forms of vibrations and accelerations transmitted through the construction to the controlled object (green color—No. 2). The last part constituted a server enabling a fast data exchange with other equipment and allowed for the connection of the keyboard and monitor for some service purpose (blue color—No. 1).

It should be emphasized that the main part of the developed stabilization system is based on the precise XSENS MTi-28A53G35 sensor module. The discussed model guarantees real-time measurement data under a drift-free 3D orientation. The features of the professional element are expressed in the Table 1. Therefore, it is an excellent device for the considered stabilization tasks.

**Figure 5:**
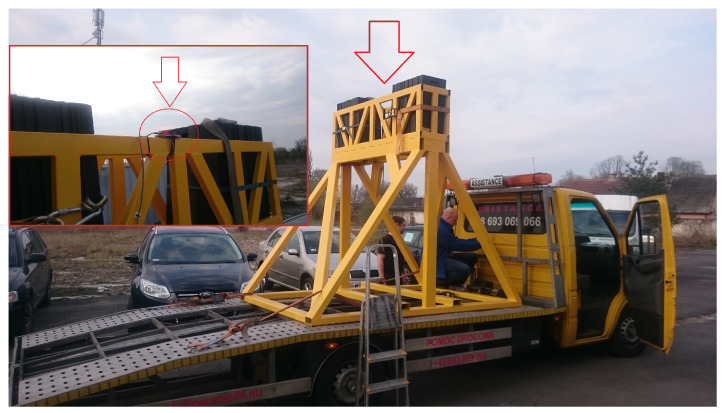
An application of the XSENS MTi-28A53G35 sensor for measurement tests [Source: MovieBird Intl.].

Figure 5 ultimately summarizes the main achievements of this section.

**Remark** **1.** *Observe that during a preparation of the physical layer, an overall calibration process was undertaken in order to simultaneously meet two optimal performance indices strictly related to accuracy and speed of the real-time system. Naturally, the discussed calibration should be understood, e.g., in the adequate time regime of the used communication protocol with respect to a maximal precision of measured data. Additionally, the transducers were calibrated according to the manufacturer’s user manuals.*

It is clear now that the resulting outcomes from the physical environment are crucial for the next stage covering the advanced control strategy design. Section 4 clearly explains this important issue.

## 4. Control System

In this section, the simulation investigations concerning the stabilization process of the Stewart platform are presented. The entire procedure was divided into different phases according to the assumed schedule of the experiment.

### Gyroscopic Measurements

**Figure 6:**
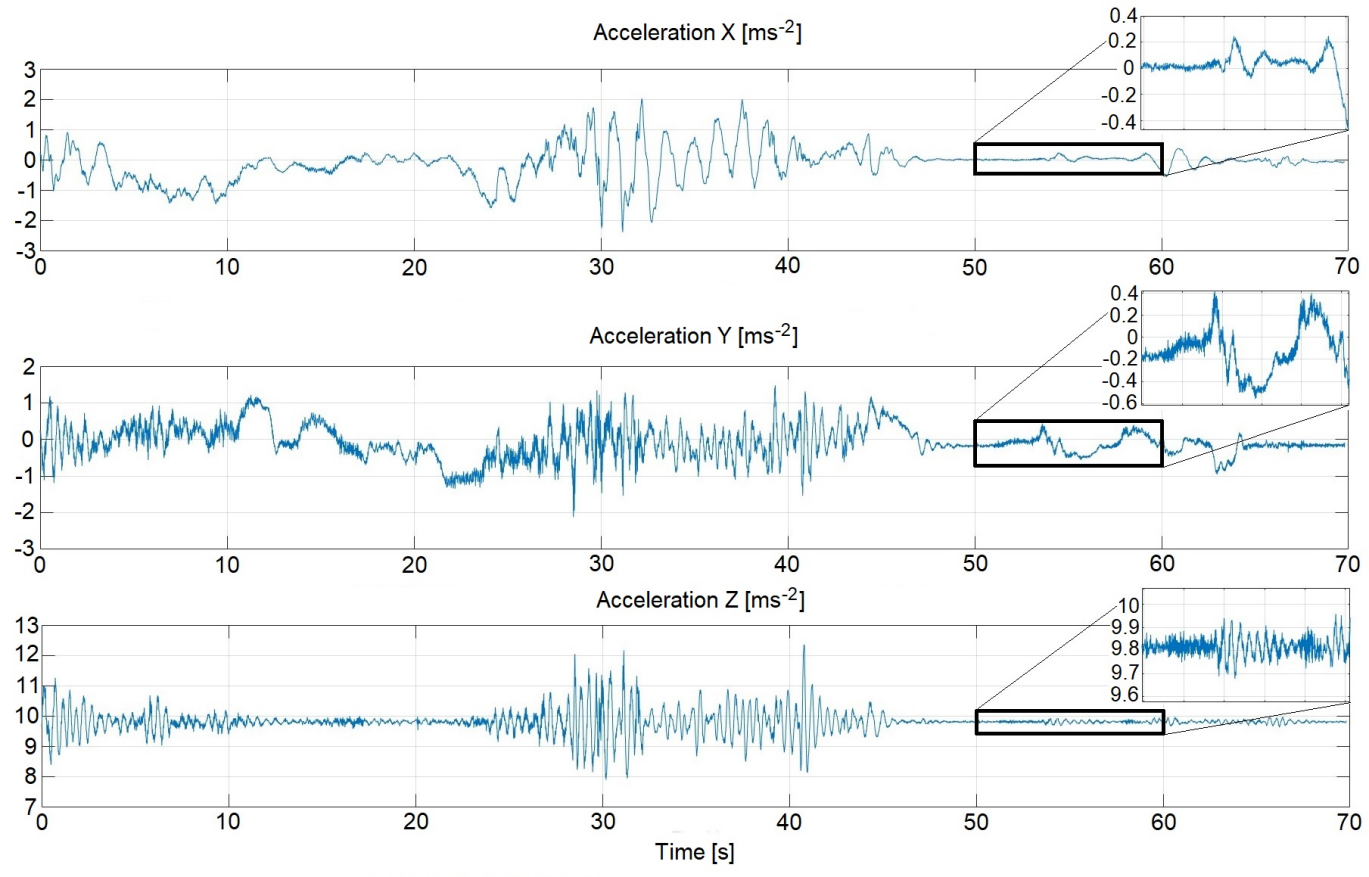
An exemplary data set [Source: MovieBird Intl.].

At the early stage of the research, the gyroscopic measurements were performed. For this reason, a truck involving the installed sensors was driven several times through one of the housing estates in Opole city. Gathered results from the accelerometer, gyroscope and magnetometer are essential for future analysis. An exemplary set of data is depicted in Figure 6.

Subsequently, the received data are helpful during estimation of the current orientation of the all measurement system. Thanks to the Matlab functions called ‘ahrsfilter(.)’ and ‘fuse(.)’, the applied solution is very convenient. Since the method employs the Kalman filter, it allows us to track the estimation error derived from the orientation, bias of the gyroscope, linear acceleration and the magnetic disturbance [35].

In order to apply the needed parameters to the Kalman filter, the data characteristics, calculated before a filtration process, were given as shown in Table 2. Moreover, the physical features of the Stewart platform were implemented into the dynamical Matlab-oriented simulation control system [36]. Thus, the whole analyzed plant can be treated as the real-life one, whose behavior was exhibited by conducted simulation studies.

**Remark** **2.** *Observe that since the theoretical and practical points of view can be slightly different in some sense, an additional set of tools had to be employed in order to fulfill the requirements of the physical environment. For that reason, the minor adjustments were adapted, covering the implementation of, for example, the window filters with different time and frequency characteristics.*

**Figure 7:**
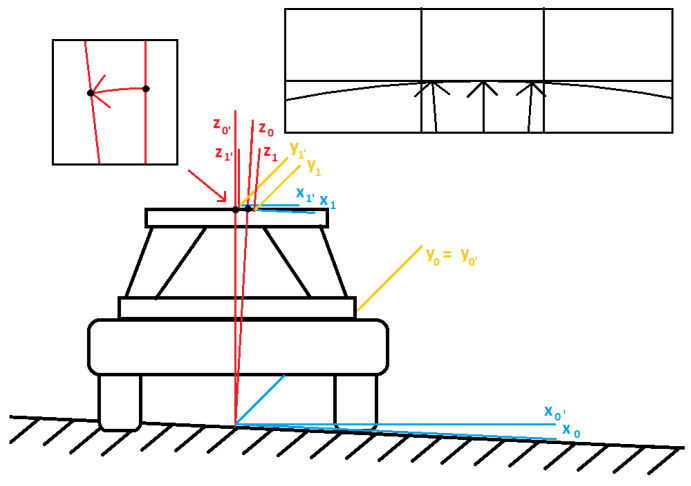
Simulation of falling into holes by a vehicle—the case of rotation around the *y* axis [Source: MovieBird Intl.].

It should be emphasized that the discussed studies, regarding the stabilization process of the Stewart platform, should respect various irregularities observed in the ground or even a number of holes through that the vehicle was driving. (see Figure 7).

It is clear now that we are dealing with changes of the positions of the appropriate coordinate systems with respect to the origin ones. According to the small deviations between the spherical trajectory-originated location of the entire plant in regard to the expected vertical vector, we omit the problem related to the translation of the *Z*-axis coordinate (see the upper right corner of the Figure 7). Hence, we only consider the rotation issue with respect to the basic axes, which can be described in the following manner.

(6) $$R=R_{x}\left(\alpha \right)R_{y}\left(\beta \right)R_{z}\left(\theta \right),$$

where

$$R_{x}\left(\alpha \right)=\left[\begin{array}{ccc} 1 & 0 & 0\\ 0 & cos(\alpha ) & -sin(\alpha )\\ 0 & sin(\alpha ) & cos(\alpha ) \end{array}\right],R_{y}\left(\beta \right)=\left[\begin{array}{ccc} cos(\beta ) & 0 & sin(\beta )\\ 0 & 1 & 0\\ -sin(\beta ) & 0 & cos(\beta ) \end{array}\right]\mathrm{and}$$

(7) $$R_{z}\left(\theta \right)=\left[\begin{array}{ccc} cos(\theta ) & -sin(\theta ) & 0\\ sin(\theta ) & cos(\theta ) & 0\\ 0 & 0 & 1 \end{array}\right].$$

**Figure 8:**
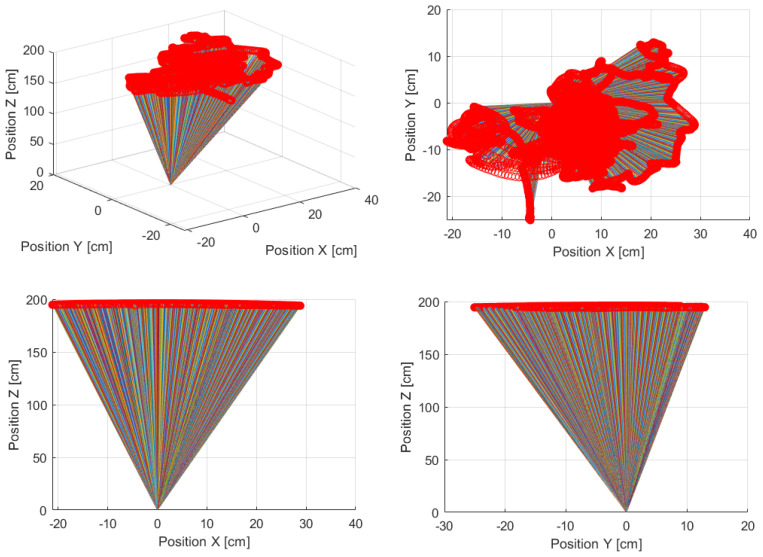
A change of the base vector orientation in the global XYZ-coordinate system [Source: MovieBird Intl.].

Referring to the above results, we can demonstrate a real change of the base vector orientation in the global XYZ-coordinate system (see Figure 8).

**Figure 9:**
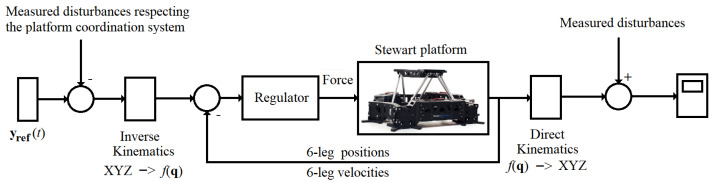
Stewart platform stabilization system [Source: MovieBird Intl.].

Next, the received data from the physical environment were treated as the disturbances in the task of the platform stabilization according to the scheme of Figure 9.

**Figure 10:**
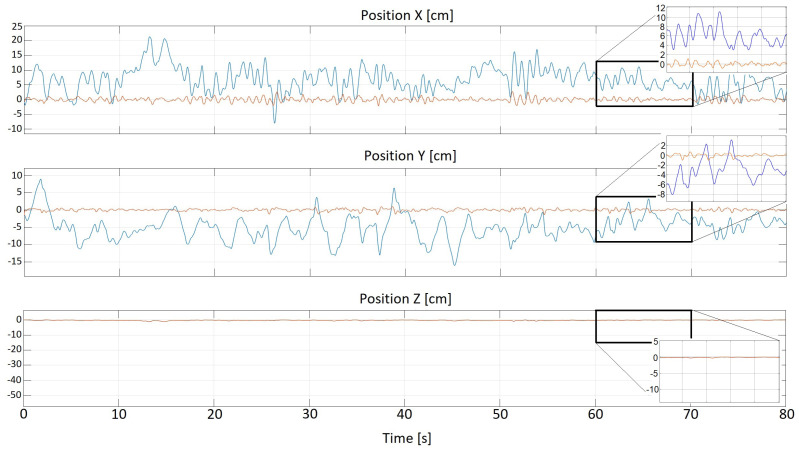
Stabilization studies—the expert method; blue line: disturbances, red line: controlled signal, speed: 10 km/h [Source: MovieBird Intl.].

**Figure 11:**
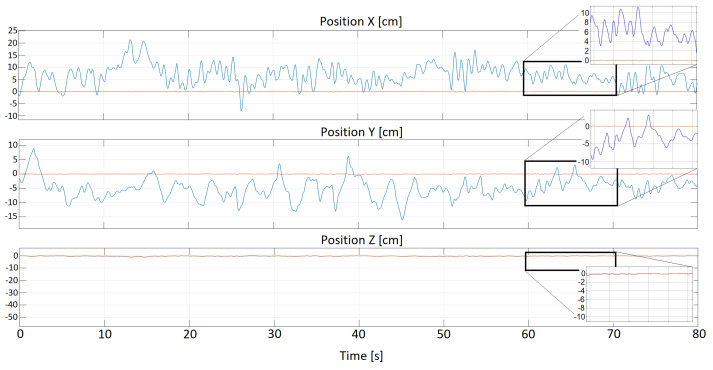
Stabilization studies—the optimization method; blue line: disturbances, red line: controlled signal, speed: 10 km/h [Source: MovieBird Intl.].

Therefore, in order to obtain a proper stabilization process, the PID controller was proposed, tuned by two methods. The first approach was based on the expert method, whereas the second optimization one applied the Gradient Descent algorithm and the Sequential Quadratic Programming. Note that simulation studies, performed in the Matlab/Simulink environment, were conducted according to the following performance index

(8) $$J=\int_{0}^{t_{s}}e^{2}\left(t\right)dt,$$

where the term $e^{2}\left(t\right)$ denotes a sum of the x(t)- and y(t)-related squared variances around the zero value, and the symbol $t_{s}$ stands for an assumed simulation time horizon. Naturally, our compensatory control scheme tends to make the performance index (8) zero. Two figures given below strictly confirm the correctness of the innovative solution. Note that the Figure 10 respects the expert method of tuning the PID parameters, whereas the Figure 11 is derived from the optimization procedure application.

**Remark** **3.** *It should be emphasized that there is no need to change the fixed operating point in a wide range since the stabilization system has only one predefined position. Naturally, due to the nonlinearity that occurred in the entire plant, in the case of a changing operating point, another optimization process should be performed.*

**Remark** **4.** *Observe that the similar solutions to these indicated in the paper can be found in the literature. A very interesting approach seems to be the method given in Ref. [37], where the roll stabilization system dedicated to a monohull ship is examined. In such a case, a set of data derived from the physical identification process was obtained in order to design two particular compensators. It turned out that the multilayer perceptron neural network-originated adaptive linear quadratic compensator outperformed the classical frequency domain-related one. This fact was confirmed by using a real ship.*

Finally, for the expert method, the criterion (8) was equal to J=8054.82. On the other hand, the optimization task brought us $J_{min}=7990.71$. Naturally, in order to meet the practical control system requirement, the triplet (x(t),y(t),e(t)) was sampled with the specified time period $T_{s}=0.01$ s. Thus, each discrete-time-oriented vector contained 8000 samples for a separate simulation study.

## 5. Conclusions and Open Problems

In the paper, the complex measurement-supported control system dedicated to the stabilization of the PS-6TM-2500 Stewart Platform is offered. For that reason, the outcomes derived from the physical tool in the form of the XSENS MTi-28A53G35 gyroscope and associated with the real parameters of the actual object structure have been engaged. Referring to the proposal, it is possible to select a linear control, which allows us to provide satisfactory results for a nonlinear plant in the context of a preserved operating point. Additionally, it turned out that the optimization-oriented approach to a design of the feedback-like control system outperformed the classical one based on the expert method. Moreover, since the synthesized plant has a high dynamic, it is impossible to adapt the convenient integration action. This fact constitutes a solid contribution to the improvement of the discussed stabilization control system. Last but not least, the developed physical layer of the entire complex object can be treated as the universal one worth engagement in other real-life solutions. A set of new peculiarities covering the measurement and control techniques strictly supports the novelty of the manuscript. Following the notions, a number of open problems could immediately be given. The first important one is focused on the application of, other than the PID-type algorithm, control laws in order to fulfill the performance index requirement. It seems that the chosen predictive regulation would be very attractive here. Secondly, the real-life load synthesis in the form of a boom with a camera is really welcomed in the near future. In the end, an adaptive neural network-based phenomenon respecting uncertainties and a system robustness improvement should also be taken into account.

## Figures and Tables

**Table 1:** Technical specifications of the XSENS MTi-28A53G35 [34].

Sensor Performance	Rotate of Turn	Acceleration	Magnetic Field
Dimensions	3 axes	3 axes	3 axes
Full scale	±300 deg/s	±50 m/$s^{2}$	±750 mGauss
Noise	0.05 deg/s/$\sqrt{\mathrm{Hz}}$	0.002 m/$s^{2}$/$\sqrt{\mathrm{Hz}}$	0.5 mGauss
Alignment error	0.1 deg	0.1 deg	0.1 deg
**Attitude and Heading**		**Value**	
Static accuracy (roll/pitch)		<0.5 deg	
Static accuracy (heading)		<1 deg	
Dynamic accuracy		2 deg RMS	
Angular resolution		0.05 deg	

**Table 2:** Measured sensor noise variances—driving a car at a speed of 10 km/h.

Gyroscope Noise Variance	Accelerometer Noise Variance	Magnetometer Noise Variance
$5.5259\times 10^{-5}$	$8.1440\times 10^{-5}$	$3.1317\times 10^{-6}$

## Data Availability

Data are contained within the article.
